# Hypoxia-Induced Invadopodia Formation Involves Activation of NHE-1 by the p90 Ribosomal S6 Kinase (p90RSK)

**DOI:** 10.1371/journal.pone.0028851

**Published:** 2011-12-27

**Authors:** Fabrice Lucien, Karine Brochu-Gaudreau, Dominique Arsenault, Kelly Harper, Claire M. Dubois

**Affiliations:** Immunology Division, Department of Pediatrics, Faculty of Medicine and Health Sciences, Université de Sherbrooke, Sherbrooke, Canada; Huntsman Cancer Institute, University of Utah, United States of America

## Abstract

The hypoxic and acidic microenvironments in tumors are strongly associated with malignant progression and metastasis, and have thus become a central issue in tumor physiology and cancer treatment. Despite this, the molecular links between acidic pH- and hypoxia-mediated cell invasion/metastasis remain mostly unresolved. One of the mechanisms that tumor cells use for tissue invasion is the generation of invadopodia, which are actin-rich invasive plasma membrane protrusions that degrade the extracellular matrix. Here, we show that hypoxia stimulates the formation of invadopodia as well as the invasive ability of cancer cells. Inhibition or shRNA-based depletion of the Na^+^/H^+^ exchanger NHE-1, along with intracellular pH monitoring by live-cell imaging, revealed that invadopodia formation is associated with alterations in cellular pH homeostasis, an event that involves activation of the Na^+^/H^+^ exchange rate by NHE-1. Further characterization indicates that hypoxia triggered the activation of the p90 ribosomal S6 kinase (p90 RSK), which resulted in invadopodia formation and site-specific phosphorylation and activation of NHE-1. This study reveals an unsuspected role of p90RSK in tumor cell invasion and establishes p90RS kinase as a link between hypoxia and the acidic microenvironment of tumors.

## Introduction

Recent research indicates that important metabolic changes occur within the tumor microenvironment and that these changes correlate with tumor progression and metastasis [1]. Hypoxia has been recognized as an important feature of solid tumors and arises presumably because of an increased metabolic demand associated with defective vascularization [2]. Hypoxia plays a critical role in various cellular events, including cell proliferation and metabolism, as well as tumor invasion and metastasis [3]. In fact, the hypoxic microenvironment of solid tumors selects for survival of aggressive, highly invasive cells that have the propensity to metastasize [4], [5]. Hypoxia also triggers an increase in the rate of glycolysis. This increase has been largely attributed to the transcriptional upregulation of the glucose transporters GLUT1 and GLUT4, and enzymes of the glycolytic pathway triggered by the hypoxia-inducible factor HIF-1 [6]. Lactate production during anaerobic glycolysis generates an excess of protons that are extruded by ion transporters and pumps resulting in acidosis of tumor microenvironment [7]. pH measurement in cancer cell lines and within tumors has revealed that the extracellular pH (pH_e_) of malignant tumor microenvironment varies from 6.2 to 6.9, whereas pH_e_ in healthy tissues is 7.2 to 7.5 [8], [9], [10], [11]. In contrast, intracellular pH (pH_i_) of cancer cells is more alkaline than in normal cells. Analogous to hypoxia, various studies have shown that alterations in pH_e_ and pH_i_ modify the phenotype of tumor cells. Acidic conditions, similar to those prevailing in many tumors, have been shown to increase transcription of VEGF [12], of IL-8 [13], [14], and to promote extracellular release/or expression/or activity of key proteases such as cathepsin B and matrix metalloproteinases (MMPs) [15]. Acidosis also amplifies *in vitro* cell invasion and *in vivo* metastasis [14], [16], [17], events inhibited by the reversal of tumor acidosis by NaHCO_3_ administration [18]. Despite the physiological and clinical significance of the relationship between pH- and hypoxia-associated cell invasion and metastasis, this question remains largely unresolved.

Sodium-proton exchangers (NHEs), sodium-dependent and -independent HCO_3_^−^/Cl^−^ exchangers, H^+^/lactate co-transporters and V-ATPase are mediators of pH homeostasis in healthy as well as cancer cells. Mammalian Na^+^/H^+^-exchangers (NHEs) are members of a family of nine related gene products (NHE1-9). They are integral membrane proteins that share up to 70% amino acid identity. The plasma membrane-type NHEs (NHE1-5) primarily catalyze the electroneutral exchange of one extracellular Na^+^ for one cytosolic H^+^. NHE-1 has an ubiquitous tissue distribution, whereas NHE2-5 have a more restricted distribution. Among these exchangers, NHE-1 is considered a main regulator of pH_i_ in cancer cells. NHE-1 activity is regulated by pH_i_ and oncogenic transformation [19], [20]. NHE-1 expression and activity have been shown to enhance the invasive capability of tumor cells through increased release and activity of MMPs and cathepsins [16], [17], [21], changes in gene expression, and regulation of the actin cytoskeleton [22], [23]. NHE-1, in breast cancer cells stimulated with EGF, has also been located at invadopodia protrusion sites where the exchanger was shown to be involved in acidification of the extracellular microenvironment, resulting in focal ECM degradation [24]. In addition to its well-known N-terminal H^+^ sensor and ion translocation function, an increasing number of studies have indicated that the C-terminal cytoplasmic tail of NHE-1 is implicated in the regulation of various cellular processes [25]. For instance, NHE-1 interacts with actin-binding proteins and that results in promotion of cytoskeletal reorganization and cell migration. In addition, phosphorylation of serine residues in the C-terminal domain, by serine/threonine kinases, may increase NHE-1 activity or promote downstream signaling events associated with NHE-1 activation. Several protein kinases have been proposed to regulate NHE-1 activity, including NIK, p90RSK and ROCK1. Among these, p90RSK has been reported to phosphorylate Ser^703^ in the C-terminal domain of NHE-1, resulting in an increased rate of Na^+^/H^+^ exchange in response to serum [26]. Whereas the role of p90RSK in the activation of NHE-1 is beginning to be understood, its implication in cell migration and invasion need to be further evaluated.

Metastatic tumor cells that actively migrate and invade surrounding tissues rely on cell membrane protrusions named invadopodia to degrade the extracellular matrix (ECM) barrier. *In vitro* and *in vivo* studies on breast cancer and melanoma progression have revealed a correlation between invadopodia formation and the potential of cell invasiveness [27], [28]. The basic molecular components involved in invadopodia formation and function are getting better defined. Invadopodia formation depends on a series of complex interactions between signal transduction molecules including members of serine/threonine and tyrosine kinase families, various classes of integrins, which provide attachment to the substratum, as well as cytoskeleton components and regulatory proteins such as N-WASP, Arp2/3, Tks5, F-actin and cortactin. Soluble and membrane-bound proteases, mostly metalloproteinases, are enriched at invadopodia sites and that correlates with the invasive potential of cancer cells [29], [30]. Invadopodia formation has been shown to be induced by few stimuli, including integrin engagement by ECM components, EGF and LPA [31], [32], [33]. Therefore, despite intensive research on invadopodia biology, little is known about specific inducers/enhancers during tumor progression.

In the present study, we showed that hypoxia stimulated invadopodia formation and cell invasion in cancer cells. Using the invasive fibrosarcoma cell line HT-1080, we observed that invadopodia formation was also associated with variations in pH_i_, an observation that was related to activation of NHE-1. We also present evidence for a hitherto unsuspected role of p90RSK that is related to its capacity to phosphorylate and activate NHE1 under hypoxic conditions and, consequently, to trigger an increase in invadopodia formation. Our results provide new insights into the relationship between hypoxia and the acidic microenvironment during the metastatic process.

## Results

### Hypoxia increases NHE-1 activity in HT-1080 cells

**Figure 1:**
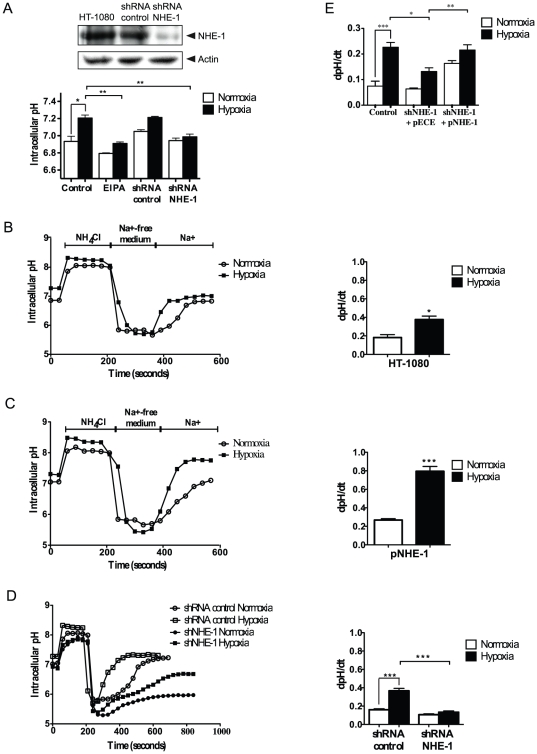
Hypoxia increases NHE-1 activity in HT-1080 cells. (A) Control (untransfected) HT-1080 cells incubated in the presence or absence of the NHE-1 inhibitor, EIPA (25 µM), or cells transfected with scrambled (control) shRNA, or NHE-1 shRNA, were incubated for 4 h under normoxic or hypoxic conditions. Basal intracellular pH (pH_i_) was measured using BCECF (n = 300 cells). Immunoblot showing knock down of NHE-1 expression in NHE-1 shRNA transfected cells. Actin was used as a loading control. (B–D) Representative traces showing pH_i_ recovery after ammonium chloride-prepulse-induced acidification in HT-1080 cells cultured under normoxic or hypoxic conditions with associated bar graph showing Na^+^/H^+^ exchange rates (n = 300 cells per condition), for (B) untransfected cells, (C) cells transfected with p-NHE-1, or (D) cells transfected with control shRNA or NHE-1 shRNA. (E) Rescue experiments were performed by transfection of NHE-1 shRNA-transfected cells with pNHE-1. Na^+^/H^+^ exchange rates were measured in cells exposed to normoxic or hypoxic conditions. Columns represent the mean ± SEM indicated by the horizontal bars. The asterisks correspond to, * *p*<0.01; ** *p*<0.001; *** *p*<0.0001.

Cytoplasmic pH (pH_i_) as well as extracellular pH (pH_e_) are tightly regulated, in part through Na^+^/H^+^ exchangers, including NHE-1, which is ubiquitously expressed in eukaryotic cells. A recent report has indicated that exposure of arterial myocytes to chronic hypoxia resulted in an alkaline shift in pH_i_ and increased NHE-1 activity [34]. To establish whether short-term hypoxia influenced pH_i_ homeostasis and NHE-1 activity in HT-1080 cells, they were exposed to normoxia or hypoxia (1% O_2_) for 4 h in a bicarbonate-free solution, a condition that prevents contributions from the Cl^−^/HCO_3_^−^ exchangers [20]. Under these conditions, steady-state pH_i_ was significantly increased in HT-1080 cells exposed to 1% O_2_ for 4 h (7.21±0. 03) as compared to cells cultured under normoxic conditions (6.93±0.06) (Figure 1A). Similar findings were observed for the 8 h time-point (data not shown). Inhibition of NHE-1 by treating the cells with the NHE-1 inhibitor EIPA or by knocking down NHE-1 using shRNA strongly reduced the increase in pH_i_ due to hypoxia (Figure 1A). In addition, NHE-1 activity (determined by the Na^+^/H^+^-exchange rate (dpHi/dt)) was increased 2-fold in HT-1080 cells cultured under hypoxic conditions when compared to cells exposed to normoxia (Figure 1B). The increase in NHE-1 activity was further augmented in cells overexpressing NHE-1 (Figure 1C). In contrast, knocking down NHE-1 fully blocked the hypoxia-mediated increase in pH_i_ recovery and Na^+^/H^+^ exchange rate (Figure 1D), an effect that was reversed by rescue experiments involving reintroduction of NHE-1 into knockdown cells (Figure 1E). We further investigated whether the findings observed in HT-1080 cells applied to other cancer cell lines. Hypoxia increased NHE-1 activity in human breast cancer cell line MDA-MB-231 and mouse B16 melanoma cells (Figure S1A–1B). Taken together, these results indicated that hypoxia altered pH_i_ homeostasis in a variety of neoplastic cells, a condition that involved the Na^+^/H^+^ exchanger NHE-1.

### Hypoxia-induced NHE-1 activity occurs in an HIF-1-independent manner

**Figure 2:**
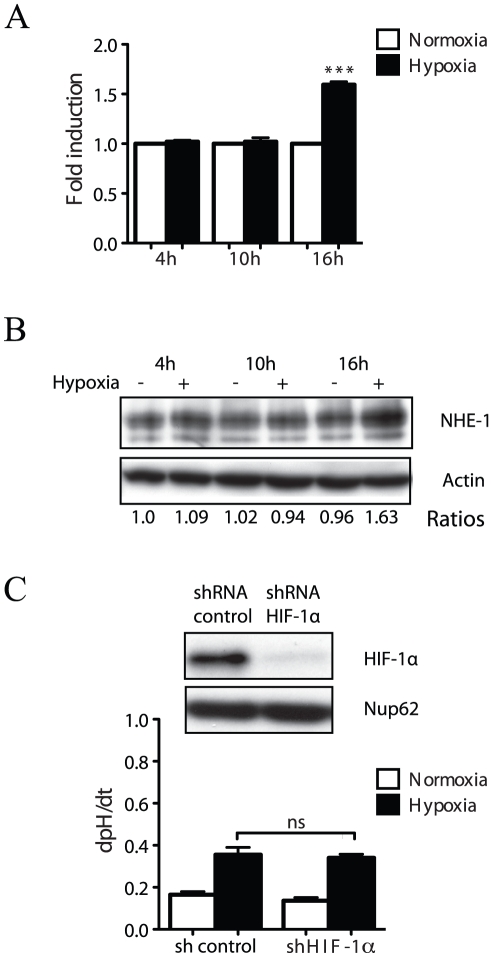
Hypoxia-induced NHE-1 activity occurs in a HIF-1-independent manner. HT-1080 cells were exposed to normoxic or hypoxic conditions for the indicated times. (A) NHE-1 mRNA expression measured by RT-PCR (n = 3). (B) Representative immunoblot showing NHE-1 expression in cell lysates (n = 3). Actin was used as a loading control. (C) Na^+^/H^+^ exchange rate in HT-1080 cells transfected with HIF-1α shRNA or control (scrambled) shRNA and exposed to 21% O_2_ or 1% O_2_ for 4 h. Immunoblot showing HIF-1α knock down in cell lysates. Nup62 was used as a loading control. NS: not significant. Columns represent the mean ± SEM indicated by the horizontal bars. The asterisks correspond to, *** *p*<0.0001.

Chronic exposure of arterial myocytes to hypoxia has been shown to result in an increased in NHE-1 expression and activity, which was proposed to be due to HIF-1-dependent-transcriptional activation of the exchanger [34]. To investigate whether our observations of an upregulation of NHE-1 activity observed after a 4 h exposure to hypoxia could be explained by a HIF-1-dependent increase in NHE-1 expression, we quantified NHE-1 mRNA and protein levels in HT-1080 cells incubated for various periods of time under hypoxic conditions. Although results showed a significant increase in NHE-1 mRNA and protein expression after 16 h exposure to a low oxygen concentration, there were no significant changes after 4 h and 10 h of exposure (Figure 2A–2B), which correspond to times where we observed an increase in NHE-1 activity (Figure 1A–1B and data not shown). These results suggested a lack of involvement of either HIF-1 and/or variations in NHE-1 concentrations in the increase in NHE-1 activity in response to short-term exposure to hypoxia. This interpretation was further supported by experiments demonstrating that the increase in the rate of Na^+^/H^+^ exchange was not affected in HIF-1 knockdown cells exposed to low oxygen concentrations for 4 h (Figure 2C). Altogether, these data indicated that the observed increase in NHE-1 activity was not due to HIF-1-dependent or -independent modulation of NHE-1 expression.

### NHE-1 is required for hypoxia-induced invadopodia formation and function

**Figure 3:**
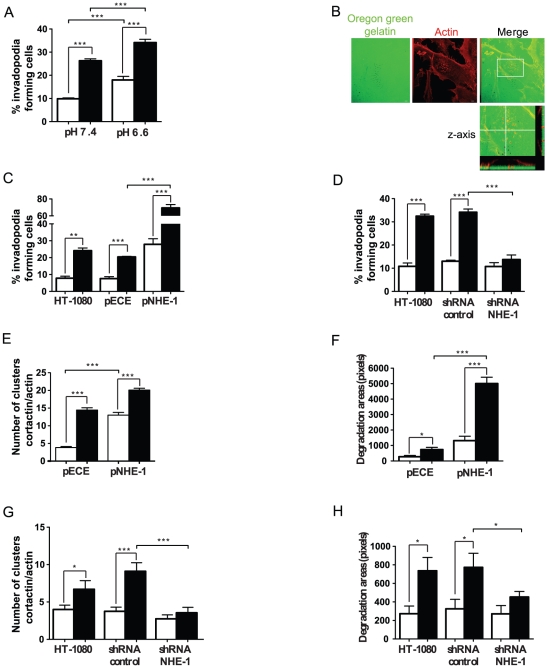
Hypoxia-induced NHE-1 activity promotes invadopodia formation. HT-1080 cells exposed to normoxic (empty bars) or hypoxic (filled bars) conditions were cultured on a layer of Oregon Green-labeled gelatin for 10 h and stained with Texas Red-phalloidin (actin) and DAPI (nucleus). The percentage of cells that formed invadopodia was determined by fluorescence microscopy. (A) Percentage of invadopodia-forming cells cultured in medium at pH 7.4 or 6.6 (n = 3). (B) Representative confocal microscopy image showing actin staining at sites of Oregon Green-gelatin degradation. Boxed area is enlarged in the corresponding lower panel. The merged image also shows a reconstruction of the x-z profile (inset) through the plane indicated by the solid line within the image. (C–D) Percentage of invadopodia-forming HT-1080 cells (C) transfected with pNHE-1 or an empty vector (pECE) (n = 3) or, (D) transfected with control (scrambled) or NHE-1 shRNA (n = 3–4). Number of invadopodia formed per cell (actin/cortactin clusters), and corresponding area of degradation per cell, quantified from 20 random fields per experiment, in cells (E–F) transfected with pNHE-1 or an empty control vector (pECE) (n = 2–3) or, (G–H) transfected with NHE-1 shRNA or control (scrambled) shRNA (n = 2–3). Columns represent the mean ± SEM indicated by the horizontal bars. The asterisks correspond to, * *p*<0.01; ** *p*<0.001; *** *p*<0.0001.

To evaluate the functional relevance of the increase in NHE-1 activity under hypoxia, we first examined whether acidic pHe influenced the capacity of the cells to degrade the extracellular matrix. HT-1080 cells were seeded onto coverslips coated with fluorescent gelatin in medium at pH 7.4 or 6.6 and the cells were allowed to degrade the gelatin matrix under normoxic or hypoxic conditions for 10 h. Cells were then fixed, stained with Texas Red-phalloidin (actin) and DAPI (nucleus), and the percentage of ECM-degrading cells was determined by fluorescence microscopy. Results showed that the ability of the cells to degrade fluorescent gelatin was increased in HT-1080 cells cultured in an acidic medium and that this was further augmented by exposure of the cells to hypoxia (Figure 3A). The presence of actin clusters at the basal plasma membrane that colocalized with areas of ECM degradation indicated that such cell degradation process was induced by invadopodia (Figure 3B). Transient overexpression of NHE-1 significantly increased the percentage of invadopodia producing cells incubated under normoxic or hypoxic conditions (Figure 3C). In contrast, knockdown of NHE-1 abrogated the capacity of the cells to produce more invadopodia under hypoxic conditions (Figure 3D). Similar results were obtained when NHE-1 activity was inhibited following cell treatment with the NHE-1-selective inhibitors EIPA or zoniporide in HT-1080, MDA-MB-283, and B16 cells (Figures S1C-S1D, S2A). Furthermore, overexpression of NHE-1 induced an increase in the number of invadopodia formed per cell, identified by clusters of actin and cortactin, two *bona fide* markers of invadopodia [35], as well as an increase in the areas of ECM degradation per cell (Figures 3E–3F). In contrast, depletion of NHE-1 by shRNA abolished both hypoxia-induced invadopodia formation and ECM degradation (Figures 3G–3H). Taken together, these results indicated that NHE-1 was required for hypoxia-induced invadopodia formation and function in cancer cells.

### NHE-1 is required for hypoxia-induced cell invasion

**Figure 4:**
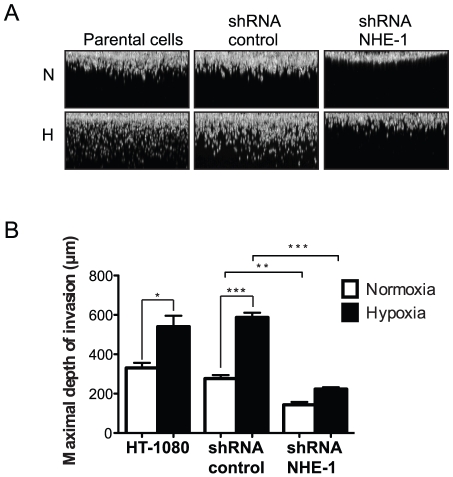
NHE-1 is involved in hypoxia-induced cell invasion. HT-1080 cells transfected with NHE-1 shRNA or control (scrambled) shRNA were allowed to invade collagen gels for 24 h under normoxia or hypoxia and stained with calcein-AM. (A) Representative confocal images showing the relative fluorescence intensity of the cells according to the depth of migration. (B) Graph represents maximal depth of invasion (n = 3). Columns represent the mean ± SEM indicated by the horizontal bars. The asterisks correspond to, * *p*<0.01; ** *p*<0.001; *** *p*<0.0001.

We next investigated the contribution of the NHE-1 exchanger to cell invasion using a 3D cell invasion assay. This assay is known to measure both the amoeboid- (protease-independent) and mesenchymal-like (protease-dependent) modes of cell dissemination [36]. Cells transfected with NHE-1 or control shRNA were seeded on top of fibrillar type I collagen gels and allowed to migrate under normoxia and hypoxia for 24 h. Cells were then stained with calcein-AM and cells that had invaded collagen gels were imaged and quantitated at each 5 µm layer within the gel using confocal microscopy. Results showed that knocking down NHE-1 significantly decreased the capacity of the cells to invade deeply into the collagen gels and fully blocked their ability to respond to hypoxia-induced enhanced migration (Figure 4A–4B). Therefore, as in the case of the invadopodia assays, our results indicated that NHE-1 was essential for hypoxia-induced cell invasion through 3D-collagen matrices. They also indicated that, under normoxia, NHE-1 was not involved in invadopodia formation but it was required for cell invasion, possibly due to its role as a plasma membrane anchor for cortical actin filaments at the leading edge of the lamellipodia [37], [38], leading to an amoeboid mode of cell migration [39].

### Activation of p90RSK by hypoxia results in NHE-1 activation and invadopodia generation

**Figure 5:**
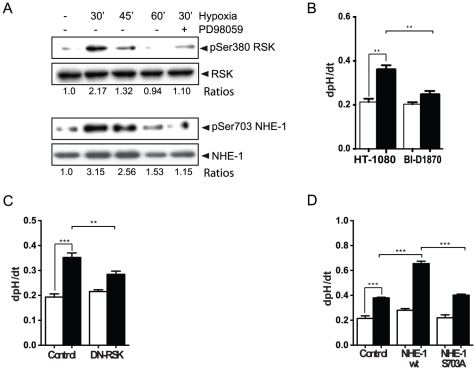
RSK-induced NHE-1 phosphorylation increases its activity in hypoxia. (A) HT-1080 cells were cultured for the indicated periods of time under normoxic or hypoxic conditions or for 30 min under hypoxia with the addition of the MEK1/2 inhibitor PD98059 (50 µM). Representative immunoblots showing p90RSK Ser^380^ phosphorylation and NHE-1 Ser^703^ phosphorylation. NHE-1 phosphorylation was revealed using antibodies directed against the 14-3-3 binding motif, following NHE-1 immunoprecipitation. Ratios of pRSK to total RSK and pNHE-1 to total NHE-1 are shown (n = 3). (B-D) Na^+^/H^+^ exchange rates were measured in cells exposed to normoxia (empty bars) or hypoxia (filled bars) in (B) the presence or absence of the p90RSK inhibitor BI-D1870 (10 µM) (n = 3), or (C) cells transfected with a dominant negative p90RSK (n = 3), or (D) cells transfected with WT NHE-1 or a S703A substitution NHE-1 mutant (n = 3). Columns represent the mean ± SEM indicated by the horizontal bars. The asterisks correspond to, ** *p*<0.001; *** *p*<0.0001.

Data shown above revealed that the increase in NHE-1 activity was not due to a HIF-1-dependent or -independent modulation of NHE-1 expression, suggesting the involvement of post-translational mechanisms. NHE-1 exchange activity can be regulated by phosphorylation of its cytoplasmic tail [25]. Among the kinases known to activate NHE-1, the serine kinase p90RSK has been shown to increase NHE-1 activity by phosphorylation of a key serine^703^ residue located in the cytoplasmic tail of the exchanger which serves as a docking site for the 14-3-3 adaptor protein [26]. To investigate whether p90RSK was involved in hypoxia-induced NHE-1 activation, we first examined the state of phosphorylation of p90RSK in HT-1080 cells under hypoxic conditions. Time-course studies revealed that p90RSK phosphorylation was sharply increased 30 min and 45 min following exposure of the cells to hypoxia, as compared to normoxia (Figure 5A). Hypoxia-induced phosphorylation was almost completely blocked by PD98059 suggesting the involvement of the MAPK pathway in p90RSK activation. In a second set of experiments, an antibody directed against the 14-3-3 binding motif was used to reveal the NHE-1 Ser^703^ phosphorylation site [40]. The levels of NHE-1 Ser^703^ phosphorylation coincided with hypoxic activation of p90RSK, suggesting a role for this kinase in NHE-1 activation (Figure 5A). To further assess the involvement of p90RSK in hypoxia-induced NHE-1 activation, HT-1080 cells were incubated in the presence or the absence of the selective p90RSK inhibitor BI-D1870 or, were transfected with a dominant negative form of p90RSK [41] prior to exposure to hypoxic conditions. Results showed that hypoxia-induced NHE-1 activity was inhibited by 60% and 40% in cells treated with BI-D1870 or in cells expressing the dominant negative form of p90RSK, respectively (Figure 5B–5C). The need for NHE-1 Ser^703^ phosphorylation in hypoxia-induced NHE-1 activation was investigated in HT-1080 cells transfected with a non-phophorylatable mutant form (S703A) of NHE-1. Results showed that cells harboring the mutant form of NHE-1 responded poorly to hypoxia, in contrast to control cells harboring wild type NHE-1 (Figure 5D) despite similar levels of overexpression (data not shown). Taken together, these results strongly suggested that phosphorylation of NHE-1 Ser^703^ by p90RSK was part of the essential events involved in the activation of NHE-1 under hypoxia.

**Figure 6:**
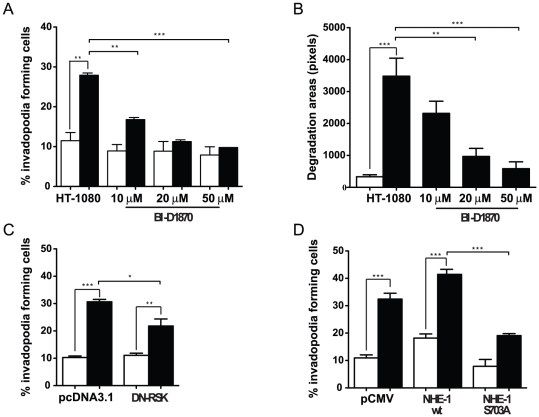
Hypoxia-induced RSK activation promotes invadopodia formation. HT-1080 cells were cultured under normoxia (empty bars) or hypoxia (filled bars). (A) Percentage of cells producing invadopodia cultured in presence or absence of BI-D1870 (n = 3). (B) Quantification of invadopodia-associated matrix degradation was performed (n = 2). (C–D) Percentage of HT-1080 cells producing invadopodia in cells (C) transfected with DN-RSK (n = 3), or (D) transfected with NHE-1 WT or S703A (n = 3). Columns represent the mean ± SEM indicated by the horizontal bars. The asterisks correspond to, * *p*<0.01; ** *p*<0.001; *** *p*<0.0001.

To evaluate the role of p90RSK in cell invasion promoted by hypoxia, we performed invadopodia formation assays in the presence or absence of inhibitors of p90RSK. Treatment of HT-1080 cells with BI-D1870 led to a dose-dependent decrease in invadopodia formation and function (area of degradation per cell) (Figure 6A–6B). A similar inhibition was observed in HT-1080 cells transfected with the dominant negative form of the kinase (Figure 6C). In addition, overexpression of the S703A NHE-1 mutant resulted in strong inhibition of HT-1080 ability to enhance invadopodia production in response to hypoxia as compared to cells transfected with wild type NHE-1 (Figure 6D). These results suggested that phosphorylation of NHE-1 by p90RSK was required, at least in part, for hypoxia-induced invadopodia formation and functions.

### NHE-1 is located at sites of invadopodia formation in hypoxic cells

**Figure 7:**
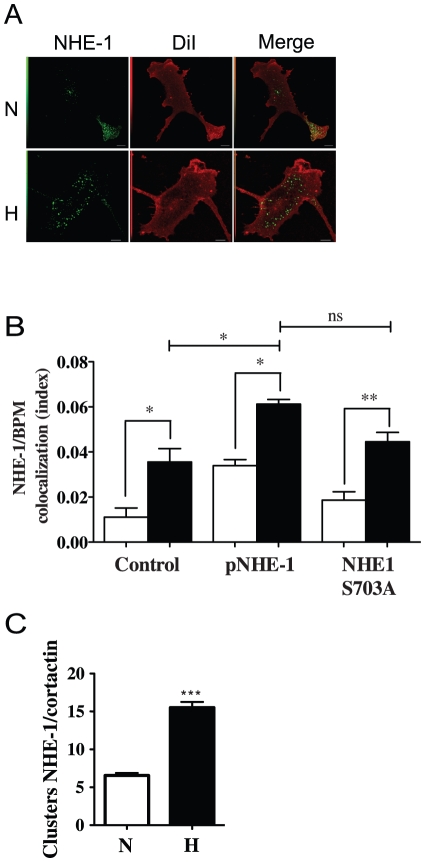
NHE-1 is localized at invadopodia sites. (A) Representative confocal microscopy images showing NHE-1 staining at the basal membrane (labeled with DiI) in HT-1080 cells cultured under normoxic or hypoxic conditions. (**B**) Colocalization index of NHE-1 and basal plasma membrane (BPM) for untransfected cells or cells transfected with WT NHE-1 or a S703A substitution NHE-1 mutant exposed to normoxic (empty bars) or hypoxic (solid bars) conditions (n = 3). (C) Numbers of cortactin/NHE-1 clusters per cell were quantified in HT-1080 cells cultured under normoxia or hypoxia for 4 h (n = 3). N, normoxia; H, hypoxia. Columns represent the mean ± SEM indicated by the horizontal bars. The asterisks correspond to, * *p*<0.01; ** *p*<0.001; *** *p*<0.0001.

We next examined whether NHE-1 was localized at invadopodia sites using antibodies that recognize the C-terminal portion of human NHE-1. Results showed that increased amounts of NHE-1 were present in numerous discrete puncta of fluorescence at the ventral surface of hypoxic HT-1080 cells (basal plasma membrane stained with DiI) that corresponded to typical invadopodia locations (Figure 7A–7B). Such increases appeared to be independent of NHE-1 phosphorylation by p90RSK because cells overexpressing the NHE-1 S703A mutant showed similar augmentations in NHE-1 localization at the basal plasma membrane in response to hypoxia compared to cells overexpressing the wild-type gene (Figure 7B). To define whether the observed areas of NHE-1 fluorescence at the basal plasma membrane corresponded to invadopodia formation sites, antibodies directed against cortactin were used. Results showed that ventral NHE-1 puncta co-localized with cortactin clusters under hypoxic conditions, suggesting NHE-1 localization to invadopodia structures in hypoxic cells (Figure 7C).

## Discussion

The tumor hypoxic and acidic microenvironment exerts profound influence on cancer cell invasion and metastasis and these characteristics are considered as important targets for the development of new anti-cancer therapies. Despite numerous studies in this field, the fundamental relationship between pH- and hypoxia-induced cell invasion is still poorly understood. Here, we showed that hypoxia promoted NHE-1 activity and its relocation to the basal plasma membrane where NHE-1-induced extracellular acidification leads to invadopodia production and cell invasion. We further provided evidence that hypoxia modulated NHE-1 activity through the activation of p90RSK.

Hypoxia is a key factor in the initiation of cancer cell invasion in solid tumors [42]. To avoid cellular acidosis, hypoxia upregulates genes involved in pH_i_ homeostasis such as CAIX and MCTs [43]. Yang et al. have recently reported that, NHE-1 activity and expression were increased by hypoxia in HepG2 cells [44]. NHE-1 contains a putative Hypoxia Response Element (HRE) in its promoter and has therefore been proposed to be a HIF-1-targeted gene [34]. In this study, we showed that the activity of NHE-1, but not its expression, was increased under hypoxia at times where we also observed an enhancement in the generation of invadopodia. Using shRNA-mediated HIF-1alpha depletion, we also showed that the increase in NHE-1 activity was independent of the transcription factor HIF-1. Although HIF-1 has been reported to be a key effector of hypoxia for cancer cell invasion, recent studies have provided evidence for the implication of other signaling pathways. Hypoxia is known to regulate the activity of the kinase mammalian target of rapamycin (mTOR), to activate the unfolded protein response (UPR) and transcription factors such as NF-kB and C/EBPß [45], [46], [47], [48]. Furthermore, HIF-2, which is also stabilized under hypoxic conditions, can interact with HREs to upregulate gene expression [49]. Unpublished results from our laboratory have indicated that in fibrosarcoma cells, hypoxia-induced increase in invadopodia production occurred in cells where HIF-1 and HIF-2 have been silenced. These findings suggest that both HIF isoforms are not necessary for NHE-1 activation and invadopodia production triggered by hypoxia.

NHE-1 activity can be regulated through post-translational modifications, including phosphorylation of serine residues in the C-terminal cytoplasmic domain. Many protein kinases regulate NHE-1 activity, including p160ROCK, NIK and p90RSK [25]. Takahashi et al. have reported that p90RSK phosphorylates NHE-1 at Ser^703^ resulting in increased activity of the exchanger in response to PDGF [26]. Here, inhibition of p90RSK or transfection with a dominant negative form of the kinase led to a strong, but incomplete, loss of NHE-1 activity (Figure 5B–5C). Similar results were observed by overexpression of a NHE-1 S703A mutant (Figure 5D), providing evidence of a direct modulation of NHE-1 by p90RSK. In addition, our results indicated an increase in p90RSK-dependent NHE-1 Ser^703^ phosphorylation following a brief (30 min) exposure of the cells to hypoxia. This observation was consistent with previous results that showed that p90RSK was rapidly activated by hypoxia in cardiac myocytes [50]. Beside p90RSK, several protein kinases can phosphorylate NHE-1 in its C-terminal tail, including p160ROCK and NIK [25]. p160ROCK is upregulated in various cancers and it has been shown to be involved in invadopodia formation [51]. ROCK is activated in hypoxia through VEGF, in a HIF-1-independent manner [52]. These data suggest that, in addition to p90RSK, the possibility exists for further regulations of NHE-1 activity under hypoxic conditions.

p90RSK has been associated with cell proliferation and cell survival [53] and recent reports have shown that p90RSK is overexpressed in human prostate and breast cancers where it is involved in cell proliferation (breast, prostate) and PSA expression (prostate) [54], [55]. Beside the emerging role of p90 RSK in cancer [56], few studies have investigated its implication in cell invasion processes. In this connection, RSK was shown to enhance the migration of melanoma cells through phosphorylation of the cytoskeletal protein filamin-A [57]. Here, we found that inhibition of p90RSK or the use of a dominant negative mutant partially reduced hypoxia- and NHE-1-induced invadopodia production. Overexpression of a NHE-1 S703A mutant also partially inhibited hypoxia-induced invadopodia formation as compared to wild-type gene. The question of which exact mechanisms regulate p90RSK activation under hypoxia is currently unknown and will be the subject of future investigations. Results presented here (Figure 5A) and from unpublished experiments have indicated that the EGFR/ERK/MAPK pathway, which is activated under hypoxic conditions [58], [59], [60], is possibly involved in p90RSK activation.

Hypoxia induced the relocalization of NHE-1 to the plasma membrane and its colocalization with the invadopodia marker cortactin. These results were consistent with a recent study indicating similar localization of NHE-1 to invadopodia in breast cancer cells stimulated with EGF where the exchanger was responsible for extracellular acidification and degradation of the ECM [24]. Despite repeated observations of NHE-1 at invadopodia, the trafficking and/or segregation mechanisms involved in NHE-1 location to these sites remain unknown. It has been reported that NHE-1 is present in lipid rafts microdomains, where it interacts with caveolin-1 [61]. Lipid rafts and caveolin-1 are required for invadopodia formation and ECM degradation by human breast cancer cells. Src, a non-receptor tyrosine kinase known to be activated by hypoxia, has been shown to regulate the localization of caveolin-1 at invadopodia formation sites [62], [63]. Therefore, the hypoxic regulation of caveolin-1 in lipid rafts may represent a potential mechanism for relocalization of NHE-1 to invadopodia.

The intrinsic ability to propagate to distant sites is the hallmark of malignant diseases and the leading cause of cancer death. Alterations in tumor cell metabolism are strongly associated with metastatic potential, which has become a central issue in cancer treatment [64]. Because of the central role of NHE-1 in pH regulation of malignant cells, it is being considered as a potential target in anti-cancer therapy. However, recent clinical studies aimed at inhibiting NHE-1 in the setting of myocardial infarction have shown disappointing efficacy with severe adverse effects [65]. Understanding the molecular mechanisms that regulate NHE-1 activity under pathological conditions is needed to develop alternative strategies to counteract the activity of the exchanger. Our findings identified p90RSK as a molecular link between NHE-1-induced extracellular acidification and invadopodia formation, providing new insights into the cooperation between hypoxia and the acidic microenvironment during cell invasion. Selective inhibition of p90RSK may be a promising approach to interfere with the metastatic potential of metabolically transformed cancer cells.

Beside cancer, the development of several hypoxia-associated diseases including pulmonary artery hypertension, myocardial infarction and ischemic retinopathies has been linked to NHE-1 activity and cardiac-specific overexpression of a dominant negative form of RSK has been shown to reduce cardiac ischemia-reperfusion injury [66], [67], [68], [69], [70]. Selective inhibition of molecular mechanisms leading to NHE-1 activation may therefore represent an alternative approach to attenuate the deleterious influence of the metabolic microenvironment in various diseases.

## Materials and Methods

### Antibodies and reagents

Antibodies used for IF microscopy or Western-blotting were purchased through commercial sources. Antibodies (and theirs dilutions) were as follows: Rabbit anti-14-3-3 binding motif, anti-RSK and anti-phosphoRSK (Ser^380^) (WB: 1/1000) were purchased from Cell Signaling (Danvers, MA). Mouse anti-tubulin (WB: 1/1000) and rabbit anti-actin (WB : 1/5000) was purchased from Sigma-Alldrich (St-Louis, MO). Mouse anti-cortactin clone 4F11 (WB: 1/1000, IF: 1/300) was purchase from Millipore (Billerica, MA). 4′,6-diamidino-2-phenylindol dilactate (DAPI), Texas Red-X phalloïdin (IF:1/200), Alexa Fluor 647-phalloïdin (IF: 1/50), and all Alexa Fluor secondary antibodies (IF: 1/200) were from Invitrogen (Eugene, OR). Goat anti-NHE1 was purchased from Santa Cruz Biotechnology (Santa-Cruz, CA). Mouse anti-NHE1 (WB: 1/500) was obtained from BD Biosciences (San Jose, CA). Anti-human NHE1 antibody N1P1 was a generous gift of Dr. Josette Noël (University of Montreal, Montreal, QC). NHE-1 inhibitors, ethyl-iso-propylamiloride (EIPA) and ConcavalinA were purchased from Sigma-Aldrich. 2′,7′-Bis-(2-carboxyethyl)-5-(and-6)-carboxyfluorescein, acetoxymethyl ester (BCECF, AM) was from Invitrogen. Control shRNA and shRNA against the 5′UTR region of NHE-1 were purchase from SABiosciences (Frederick, MD). M-Methyl-*D*-glucamine chloride (NMDG) was a kind gift of Dr. Eric Rousseau (University of Sherbrooke, Sherbrooke, QC).

### Cell cultures

HT-1080 fibrosarcoma cells (ATCC, Rockville, MD) were cultured in Minimal Essential Medium (MEM; Gibco BRL, Burlington, ON) supplemented with 10% fetal bovine serum and 40 µg/mlµ gentamycin (Sandoz, Montreal, QC) in a humidified 95% O_2_ 5% CO_2_ atmosphere at 37°C. In the case of hypoxic stimulations, cells were incubated at 37°C in an In vivo_2_ 400 hypoxic workstation (Ruskinn, Les Produits Scientifiques ESBE, Ville St-Laurent, QC) under an atmosphere of 1% O_2_ and 5% CO_2_.

### Plasmids and transfections

pECE-NHE1-GFP was a generous gift from Dr. Jacques Pouyssegur (Institute of Developmental Biology and Cancer Research, Nice, France). pCMV-NHE1-HA was a kind gift of Dr. John Orlowski (McGill University, Montreal, Canada). The plasmids pCMV-NHE1-S703A and pcDNA3.1-DN-RSK were generously provided by Dr. Bradford Berk (Aab Cardiovascular Institute, Rochester, NY). shRNA against NHE-1 was purchased from SABiosciences. Transfections were performed using the FuGENE 6 according to the manufacturer's protocol (Roche Diagnostics, Laval, QC). Stable transfectants were selected and maintained in puromycin (Cedarlane) supplemented medium.

### Measurement of NHE-1 activity

Intracellular pH (pH_i_) was measured by confocal microscopy using a fluorescent pH-sensitive probe (BCECF), as described [61]. Briefly, cells were incubated with “Na^+^ Ringer” solution containing the BCECF probe (2 µM) for 20 min at 37°C. After washing, the cells were mounted on an Olympus confocal microscope and excited at 450 nm and 488 nm. Emitted fluorescence was collected at 535 nm, at 30 s intervals. NHE-1 activity was determined by measuring the rate of pH_i_ recovery (dpHi/dt) from an acid load produced by addition of NH_4_Cl (20 mM). In the case of experiments performed under hypoxic conditions, cells were maintained under the confocal microscope equipped with a chamber containing a gas mixture of 1% O_2_ and 5% CO_2_ at 37°C.

### RNA isolation and qRT-PCR

Total cellular RNA was isolated using the TRI-Reagent protocol (Invitrogen, Carlsbad, CA) as described [71]. Quantitative real time PCR was performed using the QuantiTect SYBR Green PCR kit (Qiagen) and a Rotor-Gene 3000 instrument (Corbett Research, San Francisco, CA). Primer sequences were as follows: NHE-1 forward (5′-CCAGCTCATTGCCTTCTACC-3′), NHE-1 reverse (5′-TGTGTCTGTTGTAGGACCGC-3′), RPLPO forward (5′-GATTACACCTTCCCACTTGC-3′), RPLPO reverse (5′-CCAAATCCCATATCCTCGTCCG-3′). Each reaction was run in duplicates and values were normalized against the RPLPO housekeeping gene.

### Immunoprecipitation and Western blotting

Cells were lysed on ice in RIPA buffer. Cell lysates were centrifuged at 13,000 rpm at 4°C and protein concentration was determined using the BCA reagent (Biolynx Inc, Brockville, ON). Immunoblotting was performed as described [72]. In the case of immunoprecipitation experiments, 350 µg of total proteins were immunoprecipitated using an anti-NHE-1 antibody (dilution, 1∶100). To detect RSK-induced NHE1 phosphorylation on Ser^703^, an anti-14-3-3 binding motif antibody, was used as described [40].

### Invadopodia assays

Invadopodia assays were performed as described [32]. Briefly, cells were plated on Oregon Green- or Alexa Fluor 488-labeled gelatin and invadopodia were identified by areas of matrix degradation characterized by a loss of fluorescence. All experiments were performed at pH 7.4, unless indicated within figure legends.

### Three-dimensional invasion assays

Three-dimensional invasion assays were performed as reported [73].

### Immunofluorescence

Cells were fixed with 1% PFA for 30 min at 4°C, permeabilized with saponin (0.05% in PBS) for 20 min, and blocked in 2% BSA in PBS for 30 min. Cells were then incubated with the appropriate primary and secondary antibodies or fluorescent phalloidin, as indicated in the legends of the figures. Images were taken with a FV1000 scanning Olympus confocal microscope coupled to an inverted microscope, using a 63× oil immersion objective. To quantitate plasma membrane-located NHE-1, the basal cell surface layer (first layer in contact with matrix) was scanned and NHE-1 and plasma membrane colocalization index was calculated according to Manders *et al.* [74]. Actin/cortactin colocalization and degradation areas were determined as described [32].

### Statistical analysis

Paired or unpaired Student's *t*-test or one-way ANOVA test was used to assess statistical significance, which was set at *p*<0.05.

## Supporting Information

Figure S1 **NHE-1 is involved in hypoxia-induced extracellular acidification and invadopodia formation in MDA-MB 231 and B16 cells.** Human MDA-MB-231 breast cancer cells and mouse B16 melanoma cells were exposed to normoxic (empty columns) or hypoxic (filled columns) conditions. Na^+^/H^+^ exchange rates were measured in (A) MDA-MB-231 cells and (B) B16 cells incubated in the presence or absence of the NHE-1 inhibitor EIPA (25 µM). For invadopodia assays, cells were incubated in the presence or absence of the NHE-1 inhibitors EIPA (25 µM) or Zoniporide (100 nM) and the percentage of cells forming invadopodia was determined by fluorescence microscopy for (C) MDA-MB-231 cells and (D) B16 cells. Columns represent the mean ± SEM indicated by the horizontal bars. The asterisks correspond to, * *p*<0.01; ** *p*<0.001; *** *p*<0.0001.
(EPS)

Figure S2 **Inhibition of NHE-1 activity by EIPA reduces invadopodia formation.** HT-1080 cells exposed to normoxic (empty columns) or hypoxic (filled columns) conditions were cultured on Oregon Green gelatin for 10 h and stained with Texas Red-phalloidin (actin) and DAPI (nucleus). The percentage of cells that formed invadopodia was determined by fluorescence microscopy. (A) Percentage of invadopodia forming cells cultured in presence or absence of two different concentrations of the NHE-1 activity inhibitor EIPA (n = 3). (B) Number of invadopodia formed per cell (actin-cortactin clusters) in HT-1080 cells cultured in the presence or absence of EIPA (25 µM). Columns represent the mean ± SEM indicated by the horizontal bars. The asterisks correspond to, *** *P*<0.0001.
(EPS)
